# Desmoglein 3 acting as an upstream regulator of Rho GTPases, Rac-1/Cdc42 in the regulation of actin organisation and dynamics

**DOI:** 10.1016/j.yexcr.2012.07.002

**Published:** 2012-11-01

**Authors:** Siu Man Tsang, Louise Brown, Hanan Gadmor, Luke Gammon, Farida Fortune, Ann Wheeler, Hong Wan

**Affiliations:** a Queen Mary University of London, Barts and The London School of Medicine and Dentistry, Centre for Clinical and Diagnostic Oral Sciences, Institute of Dentistry, London, UK; b Queen Mary University of London, Barts and The London School of Medicine and Dentistry, Centre for Cutaneous Research, Blizard Institute, London, UK

**Keywords:** Desmoglein, Rho GTPases, Actin, Epithelial cells

## Abstract

Desmoglein 3 (Dsg3), a member of the desmoglein sub-family, serves as an adhesion molecule in desmosomes. Our previous study showed that overexpression of human Dsg3 in several epithelial lines induces formation of membrane protrusions, a phenotype suggestive of Rho GTPase activation. Here we examined the interaction between Dsg3 and actin in detail and showed that endogenous Dsg3 colocalises and interacts with actin, particularly the junctional actin in a Rac1-dependent manner. Ablation of Rac1 activity by dominant negative Rac1 mutant (N17Rac1) or the Rac1 specific inhibitor (NSC23766) directly disrupts the interaction between Dsg3 and actin. Assembly of the junctional actin at the cell borders is accompanied with enhanced levels of Dsg3, while inhibition of Dsg3 by RNAi results in profound changes in the organisation of actin cytoskeleton. In accordance, overexpression of Dsg3 results in a remarkable increase of Rac1 and Cdc42 activities and to a lesser extent, RhoA. The enhancements in Rho GTPases are accompanied by the pronounced actin-based membrane structures such as lamellipodia and filopodia, enhanced rate of actin turnover and cell polarisation. Together, our results reveal an important novel function for Dsg3 in promoting actin dynamics through regulating Rac1 and Cdc42 activation in epithelial cells.

## Graphical abstract

**Figure:**
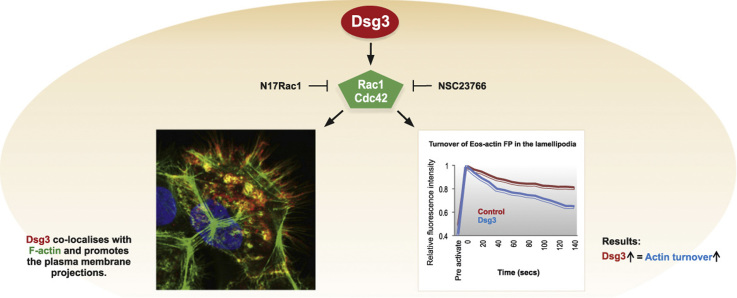

## Introduction

In the epidermis, cell–cell adhesion plays an essential role in the development and maintenance of tissue integrity [1]. The homoeostasis of epithelia requires dynamic coordination between assembly and disassembly of adhesive junctions and the underlying actin cytoskeleton [2,3]. Dysfunction of any aspects is often associated with de-differentiation and malignancy thus the precisely controlled mechanisms of cell adhesion and organisation of actin network can in addition serve as crucial regulators for other cellular processes such as polarisation, proliferation, stratification and differentiation.

Desmoglein 3 (Dsg3), a member of the desmoglein sub-family, serves as an adhesion receptor in desmosomes. It is expressed predominantly in stratified epithelia, including the basal and immediate suprabasal layers of adult epidermis and throughout the entire epithelial layers of oral mucosa. The importance of Dsg3 in cell–cell adhesion is highlighted by the autoimmune blistering disease pemphigus vulgaris (PV), where Dsg3 is the characterised autoantigen and a direct target of autoimmune antibodies [4]. Binding of the autoantibodies to Dsg3 triggers a cascade of intracellular signalling events and causes degradation and depletion of Dsg3 from the desmosomes, that in turn compromises cell to cell adhesion and disorganises the actin cytoskeleton that eventually leads to blister formation in the skin and mucous membrane. However, the molecular mechanism underlying the pathogenesis of this disease remains not completely clear [5].

Regulation of the actin cytoskeleton is fundamental to a wide range of dynamic cellular events [6]. The actin cytoskeleton provides mechanical stability and strong cell–cell adhesion based on their anchorage to adherens junctions and focal contacts [7–9], a pre-requisite for epithelial polarisation and stratification [3,10,11]. However, in response to environmental cues, rapid and localised disassembly of adhesive interactions and reorganisation of the underlying cytoskeleton allow remodelling of epithelial cells into an alternate morphology state more favourable to cell migration and wound healing. These spatial and temporal regulations of intercellular adhesions and the actin cytoskeleton often involve a wide range of cell signalling molecules such as Rho GTPases including Rac, Cdc42 and RhoA, which promote the formation of actin-cased membrane structures such as lamellipodia, filopodia, stress fibres and focal contacts [11–14].

During the early phases of calcium induced intercellular junction formation, cells project membrane protrusions such as lamellipodia and filopodia at the leading edge of cells [14]. This forward translocation mechanism helps to initiate cell–cell contacts and allows a series of transient weak adhesion zipper to form [15]. The adhesion zipper attaches the extending membrane to the extracellular matrix that is followed by clustering of adherens junction proteins such as E-cadherin, β/α-catenins along the developing cell contacts and recruits them into the punctate structures [16,17]. Concomitantly, these cadherin–catenin complexes trigger the reorganisation of actin cytoskeleton and its regulators to form productive and stable adhesion [10].

Desmosomes are another type of adhesion complex associated with the cytoskeleton of the intermediate filaments. Recent studies have shown a functional link between the desmosomal components and the actin cytoskeleton. For instance, plakophilin-1, a p120 related desmosomal plaque protein, has been demonstrated to associate with actin and is capable of inducing filopodium formation [18]. Similarly, plakophilin-2 is also reported to be involved in the regulation of Rho activity and actin organisation during intercellular junction assembly [19]. Studies based on PV-IgG have shown that loss of desmosomal adhesion in response to PV-IgG is accompanied by the re-arrangement of the junction-associated cortical actin [20], suggesting that Dsg3 might exert additional functions in regulating the actin dynamics.

Recently, we have reported that Dsg3 interacts with E-cadherin and acts as an up-stream regulator of Src activity during the formation of adherens junctions [21]. In addition, we observed that overexpression of Dsg3 promotes cell migration and membrane protrusions [22]. These findings raise a possibility that Dsg3 may play a role in regulating the actin dynamics that is responsible for the phenotype of elevated membrane protrusions and cell motility. To understand the underlying molecular mechanisms, we first examined the interaction of Dsg3 with actin and then the effect of Dsg3 modulation on the activity of the members of Rho family GTPases and the actin dynamics. Our study identified a novel role of Dsg3 in the control of actin dynamics through a mechanism of direct regulation of Rac1 and Cdc42 activation in epithelial cells. This finding will advance our understanding of the molecular mechanism underlying the pathogenesis of Pemphigus acantholysis.

## Materials and methods

### Cell culture

Two types of human epithelial cell lines, a spontaneously immortalised human skin keratinocyte line (HaCaT) and the vulva squamous carcinoma cell line (A431), were used in this study. HaCaT cells expressing high levels of Dsg3 were used as a model for loss-of-function study, while A431 cells expressing low levels of Dsg3 were used for the gain of function study. The generation of stable lines with transduction of human Dsg3 was previously described [22]. We have previously demonstrated that ectopically increased expression of Dsg3 in A431 was physiologically relevant since the Dsg3 levels were comparably lower than that of HaCaT and primary keratinocyte lines [22]. Both cell types were maintained routinely in Dulbecco's Modified Eagle Medium (DMEM) supplemented with 10% fetal calf serum (FCS). For the calcium switch assays, HaCaT cells were seeded at high density and grown in low calcium medium (EpiLife; Ca^2+^ 60 μM, Cascade Biologics) until confluent (2–3 days). They were then transferred into normal calcium medium (2 mM Ca^2+^, EpiLife) for different time periods according to the individual experiments.

### Generation of clonal cell lines

Polyclonal A431 cells with either the empty vector control (V) or overexpression of human Dsg3 (D3) were obtained before in this laboratory [22]. Limiting dilution and clonal expansion were used to select clones with the highest or lowest levels of human Dsg3 expression from A431-D3 line. A431-D3 cells were diluted and seeded ∼1 cell/well in a 96-well plate. Wells with a single clone were selected and allowed to grow for a few weeks before being transferred to big dishes for alonal expansion. The Dsg3 expression among these clones was established by Western blotting. The clones with the highest and lowest levels of Dsg3 expression such as C11 and C7 were used in the subsequent experiments.

### Transfection

A self-designed siRNA sequence (RNAi-1) specific for human Dsg3 mRNA corresponding to nucleotides 620–640 of the respective coding region (Accession: NM_001944.1)(AAATGCCACAGATGCAGATGA) along with a scrambled control (AACGATGATACATGACACGAG) were synthesised and used in our knockdown study [23]. In addition, an ON-TARGET plus SMARTpool (RNAi-2) that is commercially available (Dharmacon) also was used. It is worth noting that the nucleotides sequence 620–640 is identical to the corresponding nucleotides in the dog Dsg3 mRNA, and therefore was used in all our knockdown experiments in MDCK cells. The transfection procedures using oligofectamine or DharmaFECT reagent were described previously [21,23]. Endogenous Dsg3 was transiently knocked down at the concentrations of 50–100 nM of siRNA for 48 h by this standard protocol [23].

### Immunofluorescence and confocal microscopy

Cultured cells grown on coverslips were fixed and permeabilised with either ice-cold 1:1 methanol:acetone for 10 min or 4% formaldehyde in PBS for 10 min and then 0.1% Triton X-100 for 5 min. For the staining of Dsg3 and Phospho-Myosin Light Chain 2, MDCK cells were permeabilised with Triton buffer for 1 min instead of 5 min. Fluorescent staining was performed using the following antibodies/reagent: 5H10, mouse Ab against Dsg3 (Santa Cruz Biotechnology); AHP319, rabbit Ab against Dsg3 (Serotec); 115F, mouse Ab against desmoplakin (a generous gift from Prof. Garrod), HECD-1, mouse anti-E-cadherin (Abcam); Phospho-Myosin Light Chain 2 (Ser19) mouse mAb (Cell Signalling), Alexa Fluor 488 phalloidin (Invitrogen), Secondary Abs were Alexa Fluor 488/568/647 conjugated goat anti-mouse or anti-rabbit IgGs (Invitrogen). Images were acquired on a Zeiss Meta 510LSM laser scanning confocal microscope using a 100×/1.4 NA objective or a Leica DM5000 epifluorescence microscope using 40×/1.0 NA or 63×/1.2 NA objectives.

### Live cell imaging and image analyses

To study random migration, A431 cells were plated on glass-bottom cell culture dishes (WPI) 6 h prior to imaging. Cells were treated with or without 30 μM Rac1 inhibitor in normal growth medium for 1 h before live cell microscopy. During the experiments, cells were incubated at 37 °C in a humidified chamber at 10% CO_2_. Time lapse imaging was carried out on an inverted microscope (Zeiss) using a 20× objective and phase contrast optics. Cell images were collected every 5 min for 18 h using Metamorph (Molecular Devices). All time-lapse sequences were tracked using Metamorph as described previously [24]. To analyse cell membrane dynamics, A431 cells were seeded on glass-bottom cell culture dishes (WPI). Spinning disk confocal microscopy was used for the analysis of membrane ruffling. To label cell membranes, 1 M CFSE was added 30 min before imaging. A time-lapse series was collected every 5 s for 5 min using a 60×1.4 NA objective. Image sequences were analysed with the kymograph function in Metamorph. To analyse actin dynamics, cells were transfected with the photoconvertable EosFP-actin [25]. A small region of actin was photoconverted from GFP to RFP and the dynamics of this region were followed by collecting an image every 10 s for 10 min. Data were collected on a Zeiss 510LSM inverted microscope using a 60×1.4 NA objective. Image data were analysed as described previously [26].

The colocalisation index was quantified with ImageJ (〈http://www.macbiophotonics.ca/imagej/〉), cell height was calculated from the Z-series. The xz image panels underneath of each image are the cross sections along the green line which shows the vertical protein distribution. Student's *t*-test was used and statistical significance was accepted for *p*<0.05.

### Co-immunoprecipitation and Western blotting

For total cell extraction, cells at appropriate confluence were washed with ice cold PBS and lysed on ice with 2× sodium dodecyl sulphate (SDS) laemmli sample buffer (0.125 M Tris-Cl pH 6.8, 4% SDS, 20% Glycerol and 10% (v/v) β-mercaptoethanol). Cell lysate was immediately cleared by centrifugation at top speed for 10 min. The unused lysates were stored at −20 °C. The Triton soluble and insoluble fraction of protein was prepared as described previously [21].

For co-immunoprecipitation (co-IP), cells were washed with ice-cold PBS and then lysed in ice cold RIPA buffer (Upstate) containing 1% NP-40 and a protease-inhibitor cocktail (Calbiochem) for 10 min at 4 °C. Lysates were clarified by microcentrifugation. A given amount of total protein (usually 500–1000 μg as determined by *DC* protein assay (Bio-Rad)) was used for IP with Abs coupled with Dynabeads (Invitrogen) for 3 h before adding into the cell lysates and incubation overnight at 4 °C with rotation. Immunoprecipitates were washed thoroughly prior to resuspension in 2× Laemmli sample buffer and boiled for 3 min. Aliquots of the denatured proteins were separated by SDS-PAGE and processed for Western blotting. For co-IP experiments on Triton soluble and insoluble fractions, cells grown in 100 mm Petri dishes were subjected to protein extraction in 500 μl of Trition X-100 lysis buffer (10 mM Tris-HCl, pH 7.5, 150 mM NaCl, 2 mM ethyleneglycol-bis-(β-aminoethylether)-N,N,N′,N′-tetraacetic acid (EGTA), 5 mM ethylenediamine tetraacetic acid (EDTA), 1% Triton X-100, 1 mM phenylmethylsufonyl fluoride and protease inhibitor cocktail) for 10 min at 4 °C. After centrifugation the supernatant was denoted as the Triton X-100 soluble fraction and the un-dissolved pellet was subsequently extracted with 200 μl RIPA buffer. After centrifugation the supernatant was denoted as the Triton X-100 insoluble fraction. Protein concentration was determined by *DC* protein assay. Co-IP from each fraction was carried out as described above. For co-IPs using human skin lysates, the tissue was obtained from breast reduction following appropriate ethical approval and patient consent. The skin was washed thoroughly and cut into small pieces before incubation in 2 mg/ml dispase in DMEM for 24 h. The epidermis was separated from dermis before being transferred into 1× RIPA buffer on ice and then subjected to the routine procedures for co-IP.

For pull down assay using GTPases as baits, glutathione–agarose beads complexed with the GST fusion proteins, corresponding to the p21-binding domain (PBD) of human PAK-1 or Rhotekin, were used to pull down the active GTP-bound Rac1/Cdc42 and RhoA from A431-V and -D3 cells following the manufacturing protocol (Rac1/Cdc42 Activation Assay Kit, Millipore). SDS-PAGE sample loading buffer was used to elute the GST-fusion protein from the glutathione resin. Blots were probed with Mouse anti Cdc42, Rac or RhoA primary antibodies (Millipore) and HRP–Goat anti-mouse secondary antibodies.

## Results

### Dsg3 and actin colocalise at cell junctions

**Fig. 1:**
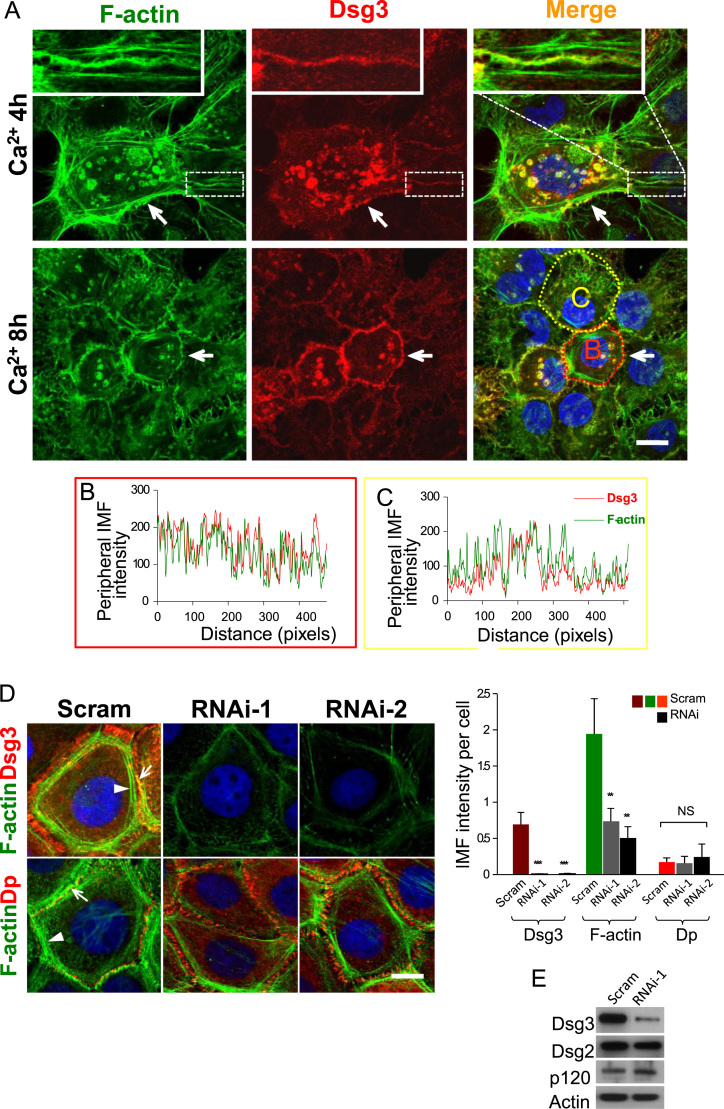
Colocalisation of Dsg3 and F-actin at the plasma membrane in normal human keratinocytes. (A) Calcium (2 mM) induced junction formation for 4 h or 8 h in HaCaT cells followed by flourescent staining with mouse anti-Dsg3 antibody (red) and A488 conjugated phalloidin (green). Arrows indicate the colocalisation of Dsg3 with F-actin, especially the junctional actin at the plasma membrane. (B,C) The profiles of the peripheral fluorescent intensities of Dsg3 and F-actin along the marked dotted lines in cells B (red) and C (yellow) showed a general trend of overlapping of two proteins. (D) HaCaT cells transiently transfected with either scrambled (Scram) or two different Dsg3 specific siRNAs (RNAi-1 and -2) at the concentration of 50 nM, for 48 h prior to calcium switch for 5 h followed by fluorescent staining for Dsg3 or desmoplakin (*Dp*, a negative control) combined with F-actin. An efficient inhibition of the Dsg3 expression was seen in both Dsg3 knockdown cells. Marked reduction of overall actin staining including both junctional actin (arrows) and cortical actin bundles (arrowheads) were observed in RNAi treated cells compared to scrambled control. Quantitation of the peripheral fluorescence intensity is shown in bar chart (mean±SEM from at least four arbitrary images in each group, **p<0.01, ***p<0.001). (E) Western blots of HaCaTs without or with Dsg3 knockdown for the indicated proteins. Scale bars, 10 μm. (For interpretation of the references to colour in this figure legend, the reader is referred to the web version of this article.)

To determine if Dsg3 colocalises with actin at cell junctions, fluorescent staining of endogenous Dsg3 and F-actin in immortalised normal keratinocyte HaCaT cells was carried out. Cells were initially grown in low calcium medium until 90% confluence. Then calcium was added in the culture medium (Ca^2+^ 2 mM) for 4 h and 8 h, respectively, to trigger junction formation prior to fluorescent staining. Confocal microscopy showed that Dsg3 co-localised with F-actin, particularly with the population of junctional actin at both time points [10] (Fig. 1A inserts). After 8 h of calcium incubation, the number of cytoplasmic vesicles containing both Dsg3 and F-actin decreased as the junctions matured (Fig. 1A). To determine the exact nature of the colocalisation between Dsg3 and F-actin at cell–cell junctions the profiles of the peripheral fluorescent intensities for Dsg3 and F-actin in two individual cells were plotted (Fig. 1B and C). The graphs showed a largely overlapping staining and parallel fluctuations of Dsg3 and F-actin at the cell junctions suggesting a close relationship between these proteins.

To follow up this study, the Dsg3 protein expression in HaCaT cells was transiently depleted using RNAi-1 and RNAi-2 for 2 days prior to a calcium switch for 5 h followed by fluorescent staining (Fig. 1D). Qualitative analysis of the peripheral Dsg3 and F-actin showed that knockdown of Dsg3 using either RNAi-1 or RNAi-2 caused a significant reduction (<70%) of the fluorescence intensity of Dsg3 and a concomitant decrease of the actin expression at cell–cell junctions (*p*<0.01, Fig. 1D and Supplementary Fig. S1). It is worth noting that control cells with elevated peripheral Dsg3 expression exhibited a clear organisation of two actin populations, *i.e*. the junctional actin and the peripheral thin actin bundles [10] (Fig. 1D arrows) suggesting progression of active adherens junction assembly. However, these two actin population appeared missing in the knockdown cells with no apparent peripheral actin bundle formation (RNAi in Fig. 1D and Supplementary Fig. S1). To validate that this observation was functionally relevant, we performed fluorescent staining of F-actin in conjunction with desmoplakin that served as a negative control in the study. As shown in Fig. 1D, no change of the peripheral fluorescence intensity of desmoplakin was seen between control and Dsg3 depleted cells. This result suggests that the cortical actin organisation is dependent upon, at least in part, the peripheral expression of Dsg3. Western blot analysis of HaCaTs treated by Dsg3 specific RNAi also demonstrated a sufficient suppression of Dsg3 and no evident change for p120 but only a marginal reduction for Dsg2 and actin (Fig. 1E).

### Association of Dsg3 with actin

**Fig. 2:**
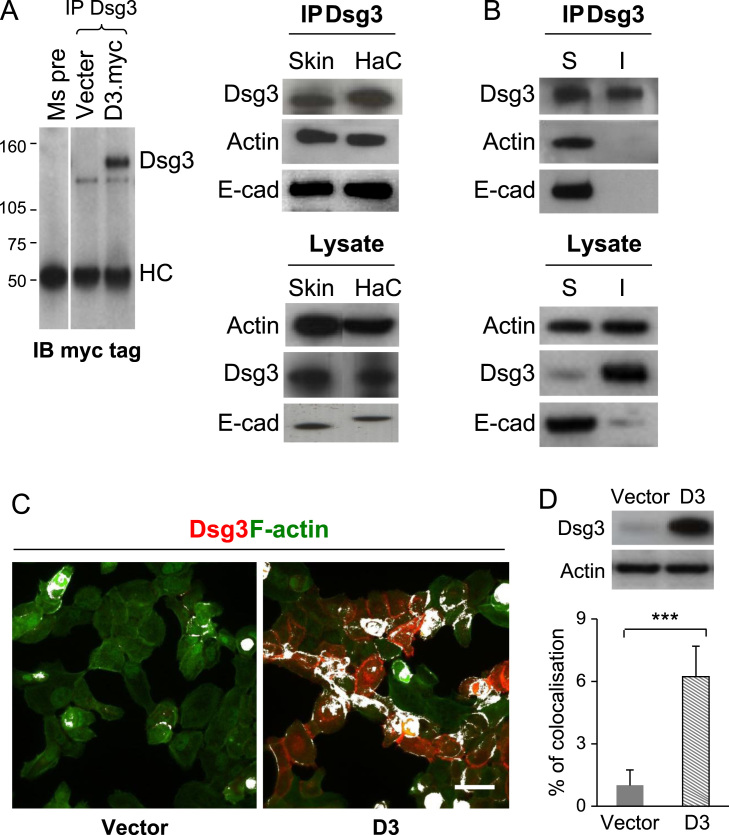
Association of Dsg3 with actin. (A) Co-immunoprecipiation of lysates of HaCaT cells and human breast skin pulled down with Dsg3 antibody. Actin was found to be co-purified with Dsg3 in both HaCaTs and human breast skin sample in addition to E-cadherin. The blots on the left were controls for co-IP pull-down with either mouse pre-immune IgG or anti-Dsg3 and blotted for myc tag. Only sample with Dsg3.myc expression displayed a positive band of Dsg3. (B) The soluble and insoluble fractions of HaCaT cells were subjected to sequential extractions using 1% Triton X-100 and RIPA buffer (1% NP-40), respectively, prior to co-IP pulled down with mouse anti-Dsg3 antibody. Actin was shown to be co-purified with Dsg3 only in the Triton soluble fraction. (C,D) Colocalisation analysis of Dsg3 and actin staining in A431 cells of vector control (V) or with overexpression of Dsg3 (D3). The pixels with the colocalisation were highlighted in white (with ImageJ) and significantly enhanced colocalisation was seen in D3 cells (mean±SEM from three arbitrary images in each group, ***p<0.001). Scale bars, 20 μm.

To determine whether Dsg3 forms a complex with actin, co-immunoprecipitation (co-IP) pull down with anti-actin antibody was performed in various cell lines as well as in human breast skin. As shown in Fig. 2A, Dsg3-actin association was detected in both HaCaT cells and human breast skin sample, but not in the pre-immune immunoprecipitate (Fig. 2A). To further identify which subcellular fraction of Dsg3 binds to actin, HaCaT cells were grown to nearly confluence prior to sequential extractions using 1% Triton X-100 and RIPA (1% NP-40) buffers for the soluble and insoluble fractions, respectively. The resulting protein lysates were co-immunoprecipitated with anti-Dsg3 antibody and analysed by Western blotting with the anti-actin antibody. Dsg3 was detected to be co-immunoprecipitated with actin along with E-cadherin only in the Triton soluble fraction (Fig. 2B).

To further define the degree of colocalisation in relation to the levels of Dsg3 expression, fluorescent staining with anti-Dsg3 antibody and phalloidin–actin was performed in cells with or without Dsg3 overexpression (Fig. 2C and D). The colocalisation index, a merge of red (Dsg3) and green (F-actin) channels highlighted in white pixels, was quantified with ImageJ. As shown in Fig. 2C and D, overexpression of Dsg3 enhanced the colocalisation of Dsg3 and actin by >50% compared to A431-V cells (*p*<0.001). Consistent results were also obtained using A431 cloned cells selected from A431-D3 population, such as C7 and C11 with the highest and lowest expression levels of Dsg3, respectively (Supplementary Fig. S2A–C). In accord, knockdown of Dsg3 in HaCaT cells reversed this effect with 80% reduction of the colocalisation compared to the cells treated with control siRNA (*p*<0.001, Supplementary Fig. S2D and E). Together these results suggest that the degree of colocalisation is highly dependent on the level of Dsg3 expression indicating their synchrony in cells.

### Dsg3 affects peripheral F-actin assembly and cell polarisation

**Fig. 3:**
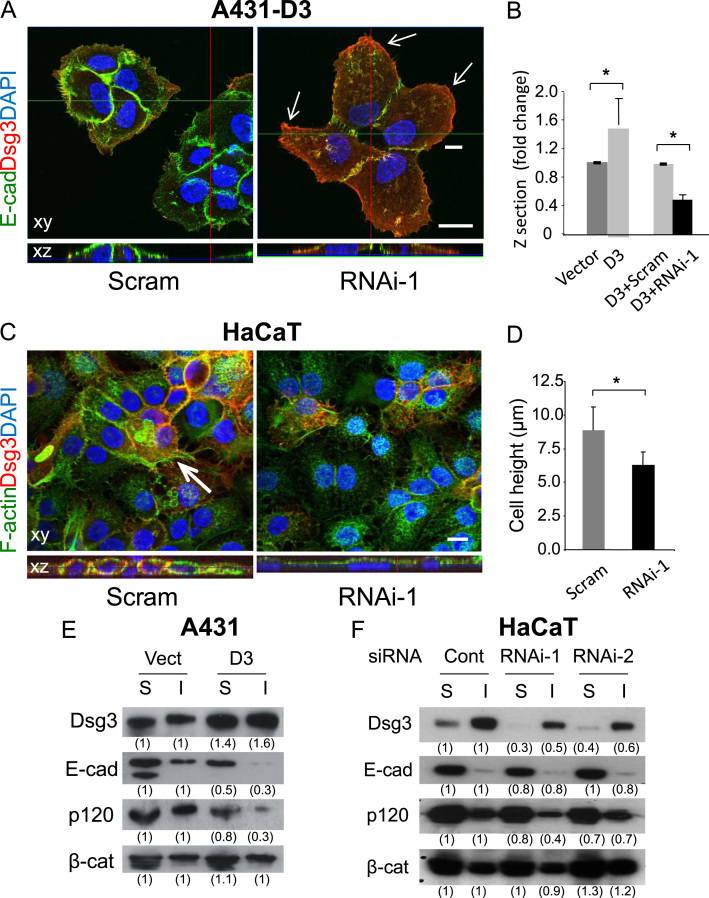
Dsg3 is required for peripheral cortical actin assembly and cell polarisation in A431 and HaCaT cells. (A,B) The representative confocal image stack of A431-D3 cells treated with scrambled or Dsg3 siRNA. Note that, comparing with the small colony with the same number of four cells in control, cells with Dsg3 knockdown appeared more flattening and showed severe disruption of E-cadherin junction formation and a marked collapse of the cell edges (arrows). Quantitation analysis in (B) showed that the enhanced cell height in A431-D3 cells (compared to vector control cells) was greatly reduced by Dsg3 knockdown (*p<0.05). Cell height was measured from five confocal Z-stacks in each group. (C,D) HaCaT cells treated with Dsg3 RNAi showed a lack of cell polarisation and apparent cell flattening with reduced cell height (*p<0.05). Sufficient knockdown was achieved in RNAi treated cells (red) with concomitantly significantly reduced intensity of F-actin staining at cell–cell junctions. Arrow in control cells (C) indicates where cells tended to stratify but this was lacking in RNAi-treated cells. The xz image panels underneath of each image are the vertical protein distribution and the shape of nuclei in RNAi-treated cells indicates the cell flattening compared to that in control. Data shown in bar charts were mean±SEM. Scale bars, 10 μm. (E,F) Western blots of the indicated proteins in Triton soluble and insoluble fractions of A431 and HaCaTs with either overexpression or suppression of Dsg3 along with the matched control, respectively. Fold changes normalised against control sample in the corresponding fractions were shown underneath of each blot. The reduced expression of both E-cadherin and p120 was seen in Dsg3 overexpressing cells, and to a lesser degree for E-cadherin in its insoluble pool and p120 in both fractions in Dsg3 knockdown cells. (For interpretation of the references to colour in this figure legend, the reader is referred to the web version of this article.)

At the adherens junctions, E-cadherin–catenin complexes are linked to the actin cytoskeleton and strong cell–cell adhesion is a pre-requisite for epithelial polarisation and morphogenesis. It has previously been shown that Dsg3 interacts with E-cadherin at cell junctions [22]. To characterise whether Dsg3 affected the E-cadherin and actin complex formation at the cell–cell contacts, RNAi mediated knockdown of Dsg3 was performed in A431-D3 and HaCaT cells for 48 h prior to fluorescent staining with anti-E-cadherin antibody and phalloidin–actin. In control siRNA treated cells, an enhanced and continuous E-cadherin staining coincident with a strong cortical junctional actin organisation was evidently seen at the cell–cell contacts (Supplementary Fig. S3). Conversely, knockdown of Dsg3 resulted in a weaker and more diffuse punctate E-cadherin and actin staining at the cell borders (Fig. 3A and Supplementary Fig. S3). In addition, cells appeared to be more flattening and a loss of stress fibres was also observed (Fig. 3A and C, Supplementary Fig. S3).

To evaluate whether knockdown of Dsg3 affected cell height, a series of confocal image stacks were collected at an interval of 0.28 μm and Z-projections were used to quantify cell height. Overexpression of Dsg3 in A431 cells caused a significant increase in cell height (*p*<0.05, Fig. 3B), while knockdown of Dsg3 reversed this effect with a significant lower cell height in both A431-D3 and HaCaT cells (*p*<0.05, Fig. 3B and D). Moreover, even within the same cell patches, cells with elevated peripheral Dsg3 expression appeared taller and showed more pronounced linear organised junctional actin than those with low Dsg3 levels where only the adjacent actin bundles next to the cell border were observed (Supplementary Fig. S4), indicating a retardation of cortical actin organisation. It is worth noting that effect of Dsg3 knockdown in A431-D3 cells on cell–cell adhesion and membrane morphology was dramatic. Cells treated with RNAi showed disruption of the E-cadherin junction integrity and a marked collapse of the leading edges as well as overall cellular architecture compared to control cells which appeared much less spreading with strong E-cadherin junction distribution (Fig. 3A arrows). Taken together these data showed that Dsg3 knockdown causes cell flattening and decreased cell height indicating a loss of contractility in cells with Dsg3 depletion. The observation of an increased cell height caused by overexpression of Dsg3 in A431 cells in Fig. 3B also suggests that Dsg3 may exert a function on cell polarisation and morphogenesis in epithelial cells through regulating the organisation of cortical actin cytoskeleton.

Our previous study based on Western blot analysis showed that overexpression of Dsg3 in A431 caused reduction of total E-cadherin expression due to Dsg3 induced Src activation. In parallel, knockdown of Dsg3 in both A431-V and -D3 cell lines restored the E-cadherin levels with no apparent change in the overall E-cadherin expression in both cell lines treated with RNAi [22]. To gain further insight, we performed Western blot analysis for E-cadherin expression in Triton soluble and insoluble fractions in A431 and HaCaT cells, respectively (Fig. 3E and F). We showed that cells with overexpression of Dsg3 exhibited reduced levels of E-cadherin, particularly in Triton insoluble fraction compared to control cells (Fig. 3E). In contrast, cells with Dsg3 knockdown showed no evident change of E-cadherin in Triton soluble fraction (the major pool of E-cadherin) but a slight reduction in Triton insoluble (minor) pool in both RNAi treated cells (Fig. 3F) suggesting a possibility of compromised E-cadherin junction assembly [21]. Reduction of p120 in both fractions was also observed in both circumstances but no change was seen for β-catenin (Fig. 3E and F).

**Fig. 4:**
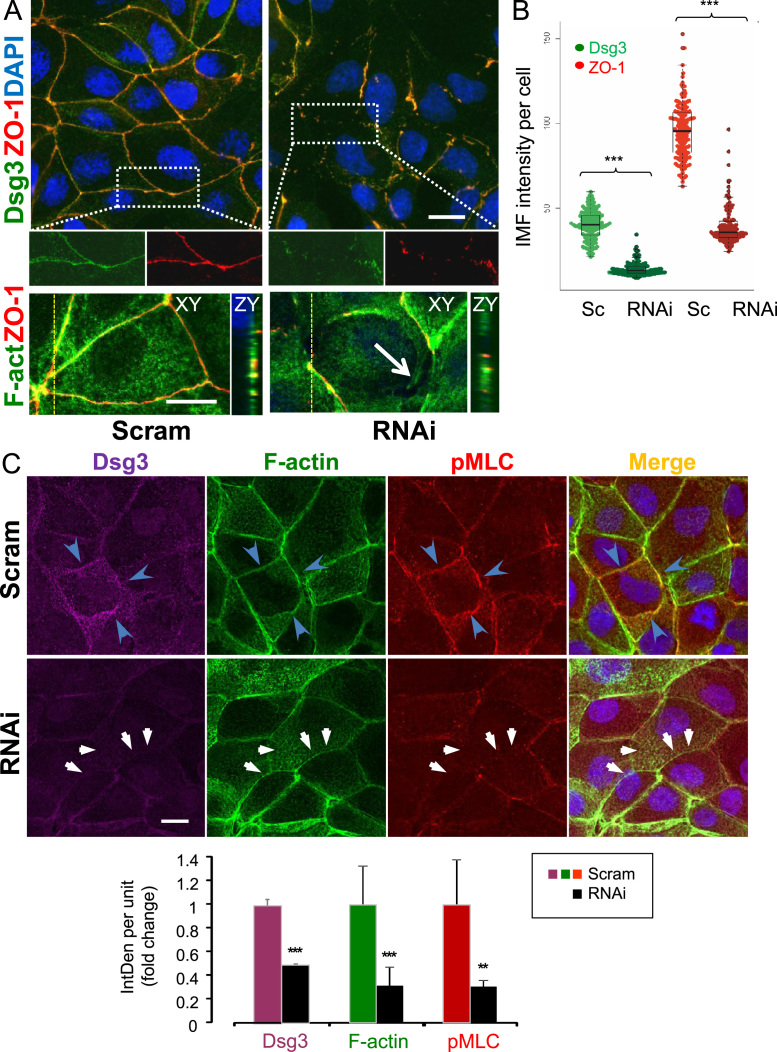
Dsg3 is required for cell polarisation and cortical actin assembly in MDCK. (A) Confocal images of Dsg3/ZO-1 or F-actin/ZO-1 double staining in MDCK cells treated with scrambled (Scram) or Dsg3 specific siRNA. Cells were permeabilised in 0.1% Triton buffer only for 1 min before fluorescent staining. The enlarged boxed areas in the panels of Dsg3/ZO-1 staining are displayed underneath. Note that Dsg3 staining appeared linear continuous, just like that of ZO-1, and both proteins were largely colocalised at the junctions. Remarkable disruption of ZO-1 was seen in cells with Dsg3 knockdown and the remaining fragmented Dsg3 was still strongly colocalised with ZO-1. The ZY sections for F-actin/ZO-1 below showed a polarised distribution of both proteins in control cells but lost in RNAi treated cells. Large intercellular gaps with concomitant loss of cortical actin bundles were indicated by arrow in RNAi treated cells. (B) Beewarm plot of Dsg3 and ZO-1 fluorescence intensity. One hundred and seventy five control cells and 130 RNAi-treated cells were analysed. Significant reduction of ZO-1 was strongly correlated with Dsg3 depletion (***p<0.001). (C) Confocal images of triple staining of Dsg3/F-actin/pMLC in scrambled control and RNAi treated cells. Again, strong correlation of three proteins at the cell borders was seen in control cells (arrowheads) but lost in RNAi treated cells (arrows). Bar chart underneath is the quantitation of the peripheral fluorescence intensities of Dsg3, F-actin and pMLC in control and RNAi treated cells (*n*>9, mean±SEM, **p<0.01, ***p<0.001). Scale bars, 10 μm.

To consolidate the finding on the role of Dsg3 in the regulation of cell polarisation, we analysed the effect of Dsg3 depletion on tight junction formation in Madin–Darby canine kidney (MDCK) cell line, a simple epithelial cell model for the study of cell polarisation and morphogenesis [28]. MDCK cells were treated with Dsg3 specific RNAi or control siRNA and were fixed with formaldehyde for 10 min and permeabilised in 0.1% Triton X-100 for 1 min. Cells were then co-stained for Dsg3 and ZO-1, the latter of which is a cytoplasmic plaque protein of the tight junctions, which is commonly used as a marker for cell polarisation in MDCK cells [29]. We found that control cells permeabilised in 0.1% Triton X-100 for 1 min gave a thin linear staining pattern for Dsg3, just like that of ZO-1, in contrast to cells permeabilised for 5 min which showed characteristic punctuate staining pattern of desmosomes that is normally observed in MDCK cells (Fig. 4A insert and data not shown). Significant knockdown of Dsg3 was detectable after 2 days of siRNA transfection (Fig. 4A and B). Of note, the Dsg3 expression at the junctions markedly overlapped with ZO-1 and cells with Dsg3 depletion displayed a remarkable loss of ZO-1 staining (Fig. 4A insert). Quantitation analysis of the Dsg3 and ZO-1 expression in over 130 cells with Dsg3 knockdown showed a strong correlation of these two proteins (*R*^2^=0.7 and Fig. 4B) indicating a Dsg3-dependent expression of ZO-1 as well as the formation of tight junction.

Double staining for F-actin and ZO-1 was also carried out. Consistently, we demonstrated a strong cortical actin organisation and ZO-1 junction assembly in control cells but a remarkable loss of ZO-1 that was accompanied with numerous intercellular gaps in Dsg3 depleted cells (Fig. 4A arrow). Detailed inspection of Z sections revealed loss of polarised distribution of ZO-1 (relative to nucleus) in RNAi treated cells compared to control cells that displayed characteristic apical ZO-1 localisation (ZY section in Fig. 4A). Consistent with our hypothesis that Dsg3 expression correlates with cell polarisation and contractility, triple immuno/fluorescent staining for Dsg3, F-actin and phospho-myosin light chain (pMLC) showed clearly that Dsg3 depletion caused a significant reduction of cortical F-actin bundles and a concomitant loss of junctional distribution of pMLC, suggesting a retardation/defect of the cellular contractivity, junction formation and cell polarisation (Fig. 4C arrows).

### Overexpression of Dsg3 enhanced membrane protrusions and dynamics

**Fig. 5:**
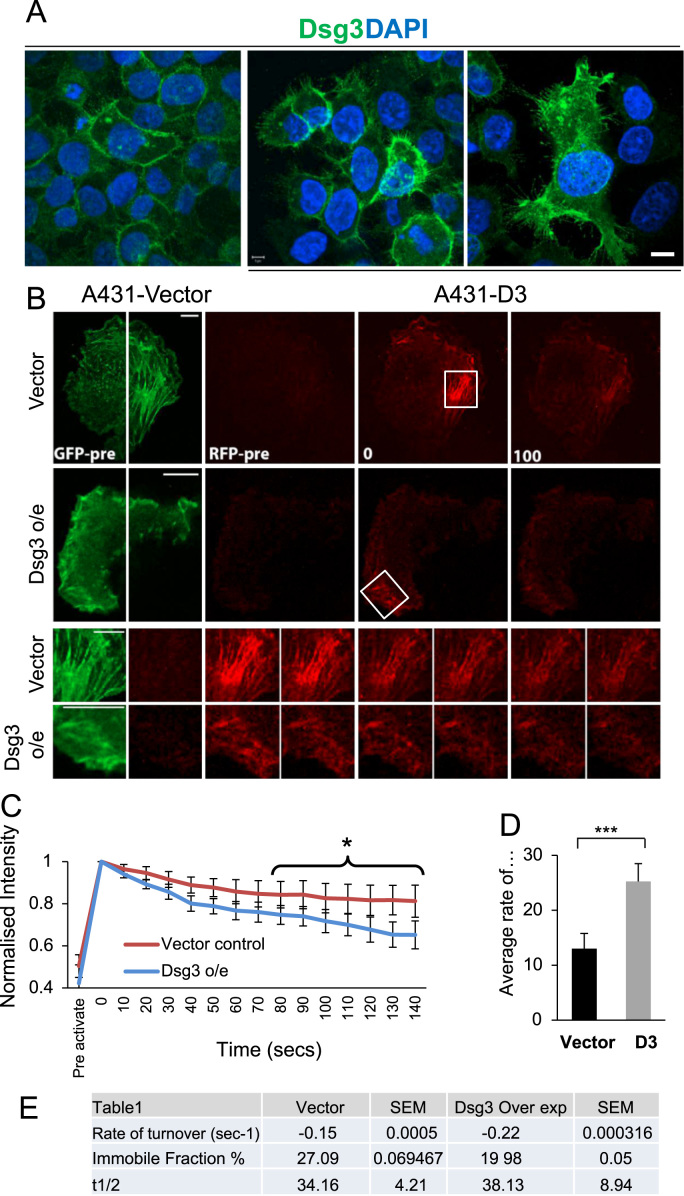
Dsg3 enhances membrane protrusions and actin dynamics in A431 cells. (A) Confocal images of A431-V (vector) and -D3 immunostained with anti-Dsg3 antibody (green) and nucleus counterstained with DAPI. (B) Confocal time-lapse series of A431 cells transfected with Eos-FP Actin. Images were acquired using 488 and 543 nm laser lines showing both the GFP and RFP species of actin. Images were acquired for 3 min, seconds after photo-conversion. Time-lapse frame rate was 1 image every 10 s. Boxes show the region of interest (ROI) photoconverted. (C) Graph showing quantification of actin dynamics in A431-V and -C7 cells following photoconversion of Eos-FP Actin from GFP to RFP over time from a representative cell. The intensity profiles for each ROI were normalised and the mean percentage recovery was measured. The time points with statistical difference were marked (*p<0.05). (D) Graph showing quantification of the rate of actin turnover following photoconversion of a small region of Eos-FP actin. Data are pooled from 20 regions of interest from 4 independent experiments (***P<0.001 using Students *t*-test). (E) Summary of the quantiation data. Scale bars, 5 μm. (For interpretation of the references to colour in this figure legend, the reader is referred to the web version of this article.)

Overexpression of Dsg3 showed striking morphological changes, resulting in the formation of enhanced membrane protrusions and ruffling in A431-D3 cells and other epithelial cells ([22] and data not shown) as compared with empty vector control cells (Fig. 5A). In addition, it was also noticed that there appeared to be a direct positive relationship between the expression level of Dsg3 and the formation of filopodia, microspikes and membrane ruffles. This observation prompted us to speculate that Dsg3 may exert a role in regulating the actin turnover directly. To investigate this question, A431-V and -D3 cells were transiently transfected with the photoconvertible probe EosFP-actin [30]. By photoconverting a small region of actin, it was possible to monitor the actin turnover and trafficking of the photoconverted actin using live cell imaging, as presented in Fig. 5B [31]. In this analysis, 4 or 5 different regions near the edge of the cells expressing EosFP-actin were photoconverted. Actin turnover was analysed by imaging cells using spinning disk confocal microscopy for 3 min. Quantitation analysis of the fluorescence intensity in the photoconverted regions showed that D3 cells had a significantly faster turnover of actin compared to V cells (*P*<0.001, Fig. 5C–E). The immobile fraction of actin in D3 cells was also reduced compared to V cells (Fig. 5E). Together, these data suggest that Dsg3 overexpression causes the actin cytoskeleton to be more dynamic near the edges of the cells. Qualitative analysis of photoconverted actin also showed that structures such as stress fibres were less stable in Dsg3 overexpressing cells as opposed to *V* cells (Fig. 5B).

### Dsg3 enhances the activity of Rac1, Cdc42 and actin turnover

**Fig. 6:**
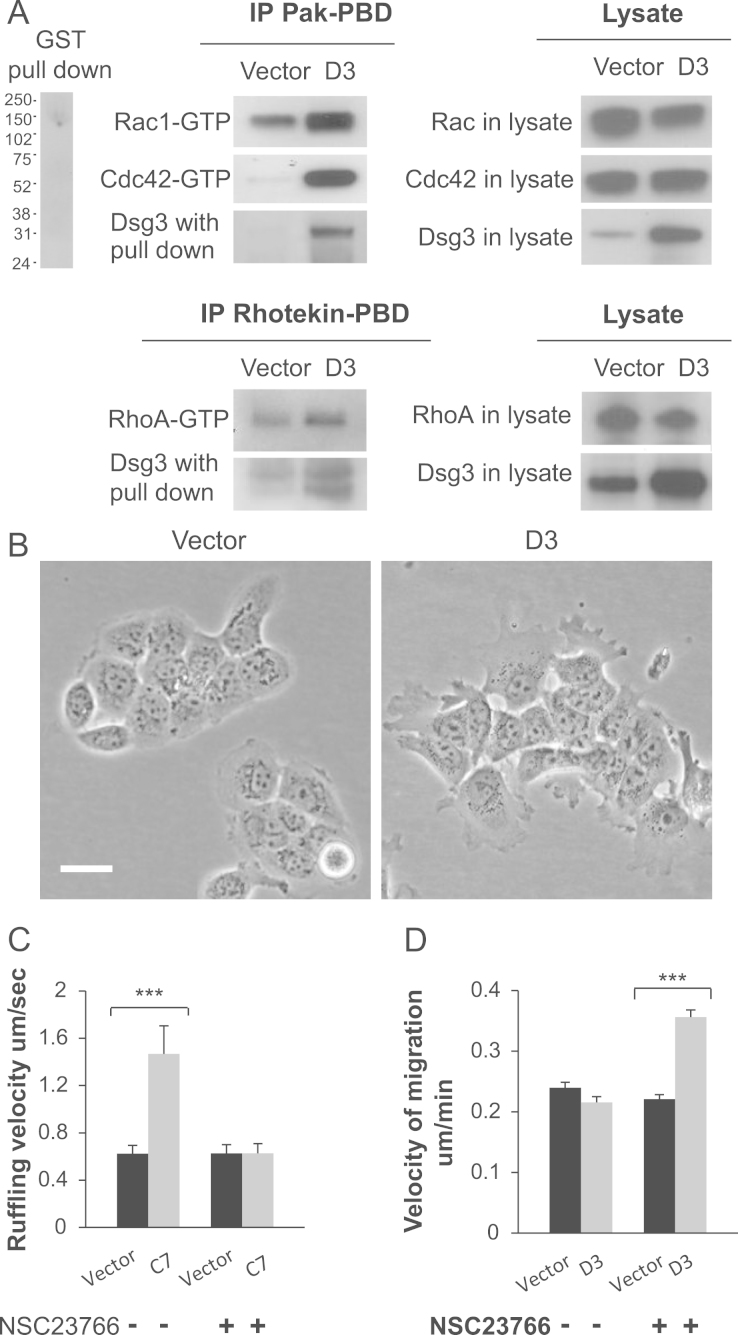
Overexpression of Dsg3 in A431 cells significantly enhances the activity of Rac1 and Cdc42. (A) Glutathione–sepharose beads complexed with GST-Pak-PBD or GST-Rhotekin-PBD fusion proteins were used to pull down the active GTP-bound Rac1 and Cdc42 or RhoA from lysates of A431-V and -D3 cells. Bead-bound complexes were loaded onto a 4–12% gradient gel and the amount of activated GTPases was determined by Western blotting with antibodies against Rac1, Cdc42 and RhoA. The negative control of GST pull down is displayed on the left. Significantly enhanced activity of Rac1 and Cdc42 but to a small extent, RhoA was detected in Dsg3 overexpressing cells. No evident changes were seen for total GTPases. Note that increased Dsg3 proteins were co-purified with the GTP-bound GTPases in D3 compared to V cells in both pull down assays. (B) Phase contrast images of A431-V and -D3 cells showing enhanced membrane projections and ruffling in D3 compared to V cells. Bar, 20 mm. (C) Graph showing ruffling velocity of A431-V and -C7 cells, treated in the absence or presence of the Rac1 inhibitor, NSC23766 at the concentration of 30 μM. Velocity measurements were obtained from kymograph analysis of the cell membrane. Overexpression of Dsg3 was shown to enhanced membrane ruffling and this could be blocked completely by Rac1 inhibitor. Data are collected from 24 regions of interest from 8 cells (pooled from 3 independent experiments, ***p<0.001). (D) Graph showing velocity of cell migration in A431-V and -D3 cells treated in the absence or presence of Rac1 inhibitor. Time-lapse series of 18 h duration were acquired at 5 min intervals however data are shown from the first 3 h of migration due to cell death in A431-D3 cells treated with NSC23766 inhibitor. Data are pooled from three independent experiments (*n*=80, ***p<0.001).

Overexpression and upregulation of Rac1 and Cdc42 have previously been shown to be associated with increased ruffling and filopodia/microspike formation [6]. To investigate if Dsg3 overexpression altered Rho GTPases activity, Rac1 and Cdc42 pull down assays were carried out using the CRIB domain from PAK fused to GST. Since inhibition of RhoA has been shown to be induced by pemphigus autoantibodies that disrupt Dsg3 and Dsg1 adhesion [32,33] we also performed RhoA pull down in parallel using the GST fused Rhotekin-PBD. Overexpression of Dsg3 was found to significantly enhance the activation of Rac1 and Cdc42 but to a lesser extent, RhoA (Fig. 6A). Of note, re-blotting the pull downs with Dsg3 antibodies showed that Dsg3 also formed a complex with GTP bound Rac1 and Cdc42 as well as RhoA, with a significant increase of the Dsg3 expression in D3 cells compared to V cells (Fig. 6A). Western blot analysis of the whole cell lysate showed no differences in total Rac1/Cdc42 content but a small reduction of RhoA in D3 cells (Fig. 6A). These findings suggest a likelihood of direct involvement of Dsg3 in the integration of Rho GTPase regulated signal pathway that controls the actin-based junction formation and epithelial morphogenesis.

Time-lapse microscopy was used to investigate the role of the Rac1/Dsg3 complex in the regulation of membrane dynamics and the actin cytoskeleton. In Dsg3 overexpressing cells, enhanced membrane protrusions (Fig. 6B) and turnover were observed consistently in A431-D3 and two cell clones, C7 and C6 that had a high Dsg3 expression (Supplementary Video 2, or data not shown). In contrast, V cells displayed much less pronounced membrane protrusions and dynamics (Fig. 6B and Video 1). To determine whether Dsg3 was required for the enhancement of membrane ruffling, we imaged A431-V and D3 cells under normal growth conditions. Spinning disk confocal microscopy was used to quantify cell membrane dynamics in V and D3 cells. Cells were filmed for 3 min using a frame rate of 1 frame every 10 s. Kymographs of active regions of cell membranes were generated to analyse the dynamics of cell membranes. These data showed that enhanced membrane ruffling occurred in D3 cells, whereas the plasma membrane of V cells was relatively inactive (*P*<0.01, Fig. 6C).

To test whether Rac1 activation is required for Dsg3-induced membrane ruffling, V and D3 cells were treated with a Rac1 specific inhibitor, NSC23766 [34] for 1 h prior to being videoed using the same conditions as above. Cells were maintained in the inhibitor for duration of the experiments. Inhibition of Rac1 strongly affected cell morphology of D3 cells by significantly reducing the membrane protrusions and ruffling to the levels similar to that of the V cells treated with inhibitor (Fig. 6C). It was also noted that Rac1 inhibited cells were less spreading out and appeared smaller compared to the untreated cells. This may suggest that Rac1 activation is important in the Dsg3-induced membrane ruffling and dynamics in A431 cells.

The observation of augmented membrane protrusions and ruffling in Dsg3 overexpressing cells led us to hypothesise that Dsg3 also involved in cell migration. To test this, V and D3 cells treated with or without the Rac1 inhibitor NSC23766 were filmed using time-lapse video microscopy for 18 h. Quantification of cell migration, using single cell tracking for 3 h, showed no obvious changes of cell migration between V and D3 cells (Fig. 6D). Interestingly, it was noted that overexpression of Dsg3 enhanced cell motility in the presence of NSC23766 initially, but this phenomenon disappeared later due to the apoptosis occurred in these cells within 3 h time period of drug treatment. However, this was not observed in the control cells which remained viable and grew normally.

### Rac1 activation is required for Dsg3 and actin interaction and colocalisation

**Fig. 7:**
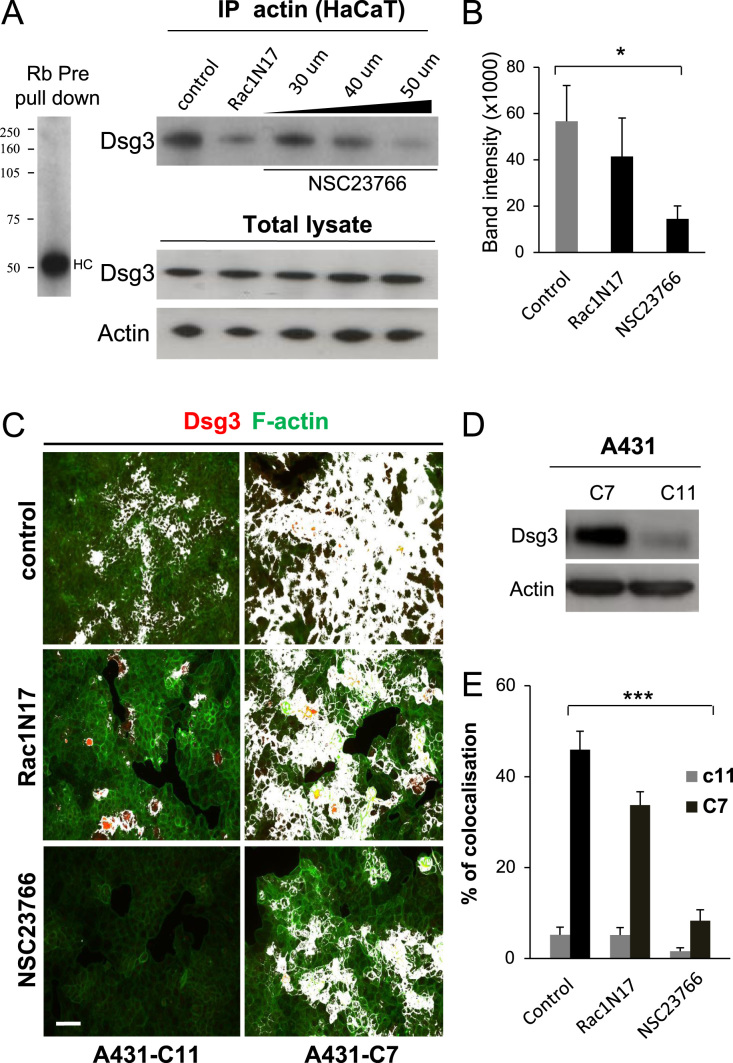
A crucial role of the Rac1 activation in Dsg3 and actin interaction. (A) Co-immunprecipitation pull down with anti-actin antibody in lysates of HaCaT cells pretreated with either the Rac1 dominant negative mutant (N17Rac1) or the Rac1 specific inhibitor (NSC23766, 30–50 μM) and Western blotting for Dsg3. Cells without any treatment were used as a control. The input of total cell lysates was shown underneath. Quantification of band densitometry from three independent experiments was shown in (B). Reduced interaction of Dsg3 and actin was observed in cells treated with both N17Rac1 and NSC23766, the latter of which exhibited inhibition in a dose-dependent manner. (C) Colocalisation analysis of Dsg3 and actin (highlighted in white) in A431-C11 (with low Dsg3 levels) and -C7 (with highest Dsg3 levels) cloned cells treated with either N17Rac1 or the Rac1 specific inhibitor, NSC23766 (30 μM). Five-seven images of confluent areas in each sample were acquired and the colocalisation was measured in ImageJ. A significant reduction of the colocalisation was particularly seen in C7 cells treated with NSC23766 (mean±SEM, ***p<0.001) and to a lesser extent in cells treated with the dominant negative mutant, N17Rac1. The Dsg3 Western blot in C11 and C7 cells was shown in (E). Scale bar, 50 μm.

The results presented above showed that Rac1 activity was substantially upregulated in the D3 cells hence, there is a possibility that Rac1 might control the Dsg3/actin interaction and colocalisation. To determine whether this was the case, the effect of Rac1 mutants and Rac1 inhibitor on Dsg3/actin interaction and colocalisation were investigated. Co-IP of Dsg3 with anti-actin antibodies was performed in HaCaT cells. In agreement with the finding in Fig. 2A, the control cells again showed that Dsg3 and actin form a complex with one another (Fig. 7A). Inhibition of Rac1 either by transfecting cells with the dominant negative N17Rac1 mutant [35], or the Rac1 inhibitor NSC23766 greatly reduced the interaction between Dsg3 and actin (Fig. 7A and B). By titrating the dose of the Rac1 inhibitor NSC23766 at the concentration of 30–50 μM for 6 h, it was possible to determine that Rac1 activity was required for the formation of the actin/Dsg3 complex (Fig. 7A and B). Confocal imaging of HaCaTs treated with the Rac inhibitor NSC23766 for 6 h showed more punctuate immunostaining and diffuse cytoplasmic distribution of Dsg3 as compared to control cells without drug treatment, indicating a disruption of junctional translocation of Dsg3 in the presence of Rac inhibitor (Supplementary Fig. S5). In addition, the reorganisation of junctional actin during the process of cell polarisation and stratification appeared to be disturbed by the inhibition of Rac1 (Supplementary Fig. S5). Together, these results show clearly that Rac1 activity is essential in Dsg3-actin complex formation and Dsg3 mediated cortical actin organisation.

We next performed fluorescent staining with anti-Dsg3 antibody and phalloidin-actin in A431 cloned cells expressing high (C7) and low (C11) levels of Dsg3, pre-treated either with Rac1 mutant or Rac1 inhibitor. The percentage of colocalisation highlighted in white was quantified with ImageJ. The representative images obtained using a fluorescence microscope showed that the colocalisation of Dsg3 and F-actin was greatly reduced by N17Rac1 mutant and NSC23766, respectively (Fig. 7C, D; *n*=6, *p*<0.001). These results demonstrated that Dsg3 and actin interaction is dependent on Rac activity and Dsg3 is also capable of directly activating Rac1. Taken together, these results uncover that Dsg3 and actin are the components of a novel signalling complex that affects the formation of cell–cell junctions and the organisation and dynamics of the actin cytoskeleton in filopodia and lamellipodia of cells.

## Discussion

In this study we aimed to understand how Dsg3 regulates membrane morphology and actin dynamics in epithelial cells. We discovered that endogenous Dsg3 colocalises and physically interacts with F-actin, particularly the population of junctional actin in a Rac1-dependent manner. Besides, overexpression of Dsg3 results in an overall increase of the Rac1 and CDC42 activities. These alterations in Rho GTPases are accompanied by pronounced membrane protrusions and dynamics, enhanced rate of actin turnover and cell polarity.

Our results showed for the first time a physical association between Dsg3 and actin and these two proteins form a complex with Rac1 which is important in the regulation of the cortical actin cytoskeleton. We demonstrated that endogenous Dsg3 and actin can be immunoprecipitated as a complex both in cultured cell lines in vitro and in human skin specimen. These two proteins are partially colocalised at the plasma membrane. Our result indicates that such an interaction requires the activation of Rac1 since inhibition of Rac1, either by dominant negative mutant or by Rac1 specific inhibitor, can abrogate the interaction remarkably. The biological relevance of this interaction is supported by the observation that the junctional actin was severely affected in pemphigus vulgaris, an autoimmune blistering disease involving circulating autoantibodies targeting Dsg3 of skin and oral mucous membrane with [20] (our own unpublished data). Furthermore, we speculate that this interaction occurs outside of the desmosomes since Dsg3 was co-immunoprecipitated with actin only in the Triton soluble fraction.

It has previously been demonstrated that loss of desmosome components such as desmoplakin resulted in defects in the maturation of adherens junctions and organisation of actin cytoskeleton [36]. Here, we showed that RNAi mediated Dsg3 knockdown resulted in a more diffuse and punctate actin staining and prevented the clustering of membrane proteins such as E-cadherin along the developing cell–cell contacts. Frequently, we saw that in small colonies, cells with severe Dsg3 depletion displayed disruption of the cell–cell junction integrity. Loss of Dsg3 caused interruption of the continuous sealing between the neighbouring cells and this was especially the case at the edge of the colonies (RNAi-1 in Fig. 3A). E-cadherin mediated junction assembly and cortical actin organisation are closely related events at the early stages of cell–cell contacts, which is essential for the proper formation of adherens junctions and desmosomes. Rho-family GTPases including Rac, Cdc42 and RhoA play a crucial role in these processes through directly regulating the organisation of the actin cytoskeleton. Inhibition of Rac1 and RhoA by pemphigus autoantibodies targeting Dsg3 and Dsg1 showed disruption of cell–cell adhesion and blister formation in epidermis [32,33,37]. Our findings in the present study suggest that Dsg3 may exert a function in engaging E-cadherin junction assembly at the early stage of cell–cell contacts. We showed that cells (in sparse culture) treated with Dsg3 RNAi exhibited defects in E-cadherin clustering and its localisation at the junctions. In addition, a concomitant reduction of cortical actin bundles and the pMLC expression were observed, rendering the collapse of cellular architecture (Fig. 3A). These phenotypic changes resemble that of the RhoA-null keratincoytes with increased cell spreading due to impaired RhoA-ROCK-MLC-mediated cell contraction [38].

Our results with ZO-1 showed that depletion of Dsg3 not only affected the adherens junctions, but also the tight junctions (Fig. 4A and B). Similarly, loss of pMLC at the junctions is observed in cells where Dsg3 is knocked down. This phenotype is likely due to the failure of junction assembly which is dependent on an interaction between E-cadherin and Dsg3, as shown in our previous work [22], and also the activity of RhoA [38]. Thus the observed alteration of the E-cadherin expression at the early stages of junction formation is likely due to the functional loss of Dsg3 rather than simply weakening of cell adhesion in general. Taken together, our results are consistent with the notion that the non-junctional pool of Dsg3 functions outside of the desmosomes and plays a signalling role in the regulation of E-cadherin junction assembly and cortical actin organisation through activation of Rho GTPases. In addition, our studies also support a general conclusion that desmosomes, adherens junctions and the actin cytoskeleton are mutually dependent and act synergistically to form and strengthen cell–cell adhesion.

Overexpression of Dsg3 elicited striking morphological changes, resulting in the formation of enhanced protrusions and membrane ruffles, prominently observable in A431-derived cells as well as other epithelial cell lines with overexpression of Dsg3 (Fig. 5A). Live cell imaging showed enhanced non-directionally protrusive activity throughout the whole cell perimeter in A431 cells with overexpression of Dsg3 as compared with control cells, a phenotype strongly suggestive of Rac1 and Cdc42 activation (Supplementary Videos 1 and 2). Indeed, our findings revealed that overexpression of Dsg3 significantly enhanced the activity of Rac1 and Cdc42, as shown by the PAK-GST pull down assay (Fig. 6A). The notion that Dsg3 regulates the organisation of actin dynamics is further corroborated by the fact that overexpression of Dsg3 considerably increased the rate of actin turnover (Fig. 5C–E). While the ability of active Rac1 and Cdc42 to produce enhanced cell protrusions and ruffling has been well documented in the literature [2,6], the upstream signal regulators activating Rac1 and Cdc42 in the presence of cell–cell contacts remained unclear. Our results extend this concept to reason that Dsg3 would be a possible candidate as the activator of Rac1/Cdc42 as well as involving in the PI3-kinase-independent activation of Rac1 by E-cadherin [39]. Furthermore, the enhancement of membrane protrusions was accompanied with the changes in the domains of plasma membrane, pointing toward cell polarity. Here, we show that overexpression of Dsg3 resulted in cell heightening, while silencing reversed this effect in both A431 and HaCaT cells, suggesting that Dsg3 could further exert its influence on cell polarity by modulating the downstream signalling events of Rac1/Cdc42 [40], the effect also observed in MDCK cells (Fig. 4). The polarisation of epithelial cells is driven by differentiation pathway and is correlated with an inhibition of cell proliferation and growth arrest. In line with this notion, our previous studies have demonstrated that primary keratinocytes with high Dsg3 levels exhibited shortened life span and underwent terminal differentiation much faster than those with low Dsg3 expression [41,42].

We noticed that inhibition of Rac activation by NSC23766 enhanced cell migration and caused early cell death in A431 derived Dsg3 overexpressing cells. This phenomenon is intriguing and perhaps involves the activation of caspase-8. There is evidence suggesting that caspase-8, which binds directly to Src, has dual roles in promoting cell migration and apoptosis depending on the protein localisations [43]. This will be an interesting avenue of investigation for the future. Unfortunately it was not possible to follow cell migration studies further because inhibition of Rac1 activity caused cell death in Dsg3 overexpressing cells. We would anticipate that analysis of V and D3 cells may show differences in cell migration if it were possible to make measurements over a longer time course. Indeed, enhanced cell migration in Dsg3 overexpressing cells has been previously demonstrated in our scratch wounding assay [22] as well as in the transwell cell migration and organotypic cell invasion assays (Wan, unpublished data).

The precise role of Dsg3 in regulating organisation of the actin cytoskeleton is not yet completely understood. Nevertheless, there is a possibility that Dsg3 may function as a bridge between actin and cadherin–catenin complexes at the plasma membrane. In support with this notion, we have demonstrated that the organisation of adherens junctions and the actin cytoskeleton was severed affected by Dsg3 silencing ([21] and this study). Therefore, our hypothesis is in agreement with Vasioukhin et al. [36], who suggested that desmosomes and their constitutive proteins are involved, at least in part, in stabilising the cytoskeletal anchorage of actin and cadherin–catenin complexes. Further studies on actin linkage to Dsg3 are required to resolve the mechanism(s) involved.

In summary, our findings suggest that Dsg3 can interact with the actin cytoskeleton and promote its organisation through regulating Rac1/Cdc42 activities in epithelial cells and that this crosstalk may play an important role in determining the cellular polarisation, bio-morphogenesis and tissue remodelling.

## Figures and Tables

**Video S1:**
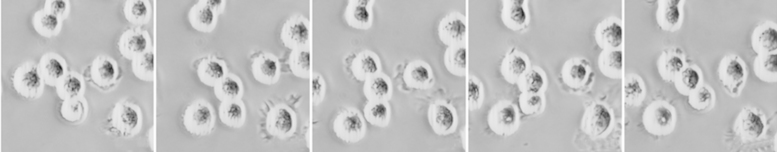
– Time lapse video of A431-V cells plated in the uncoated culture dish for 3 h before live cell imaging. Image series of 100 frames with 1.5 h duration at 1 minute interval were acquired and fast played at 120 times. doi:10.1016/j.yexcr.2012.07.002.

**Video S2:**
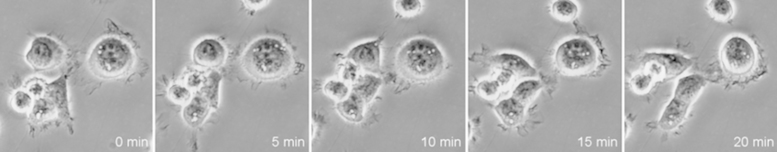
– Time lapse video of A431-C7 cloned cells plated in the uncoated culture dish for 3 h before live cell imaging. Image series of 100 frames with 1.5 h duration at 1 min interval were acquired and fast played at 120 times. doi:10.1016/j.yexcr.2012.07.002.

## Appendix A Supplementary materials

Supplementry material

## Footnotes

Appendix A Supplementary data associated with this article can be found in the online version at doi:10.1016/j.yexcr.2012.07.002.
