# Supplementary material for: Desmoglein 3 acting as an upstream regulator of Rho GTPases, Rac-1/Cdc42 in the regulation of actin organisation and dynamics
Source: Exp Cell Res. 2012 Nov 1;318(18):2269–83. doi: 10.1016/j.yexcr.2012.07.002 (PMC4022105; doi:10.1016/j.yexcr.2012.07.002)
Supplement: Supplementary file 1 — Supplementry material [file mmc1.pdf]

Supplementary Fig. S1

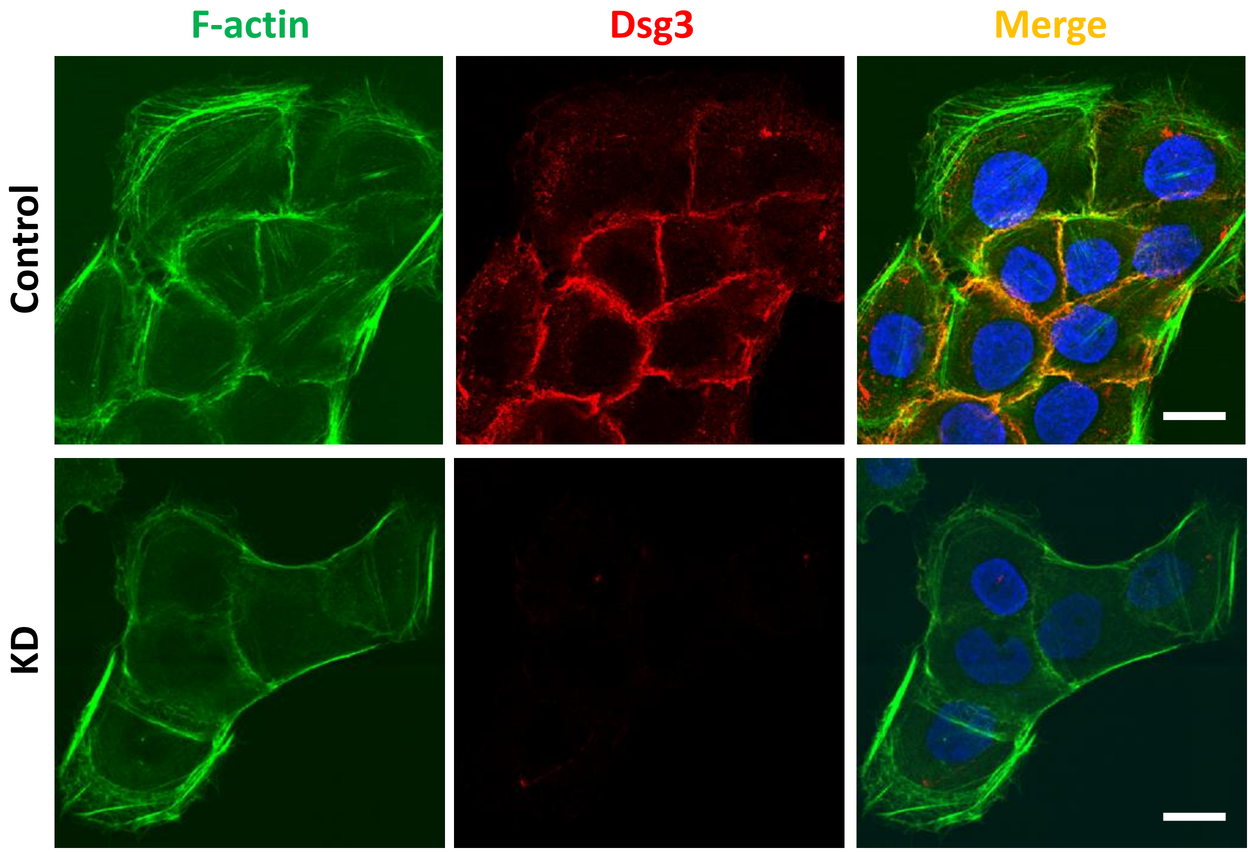

Supplementary Fig. S1 – Inhibition of Dsg3 expression affected cortical F-actin in HaCaT keratinocytes. HaCaT cells were transiently transfected with either scrambled control or Dsg3 specific siRNA for 48h before immunofluorescent staining for Dsg3 (red) and F-actin (green). An efficient Dsg3 inhibition was seen in cells with Dsg3 knockdown (KD) and a marked reduction of cortical F-actin bundles were seen in KD cells as compared with that in control cells. Scale bars, 10um.

Supplementary Fig. S2

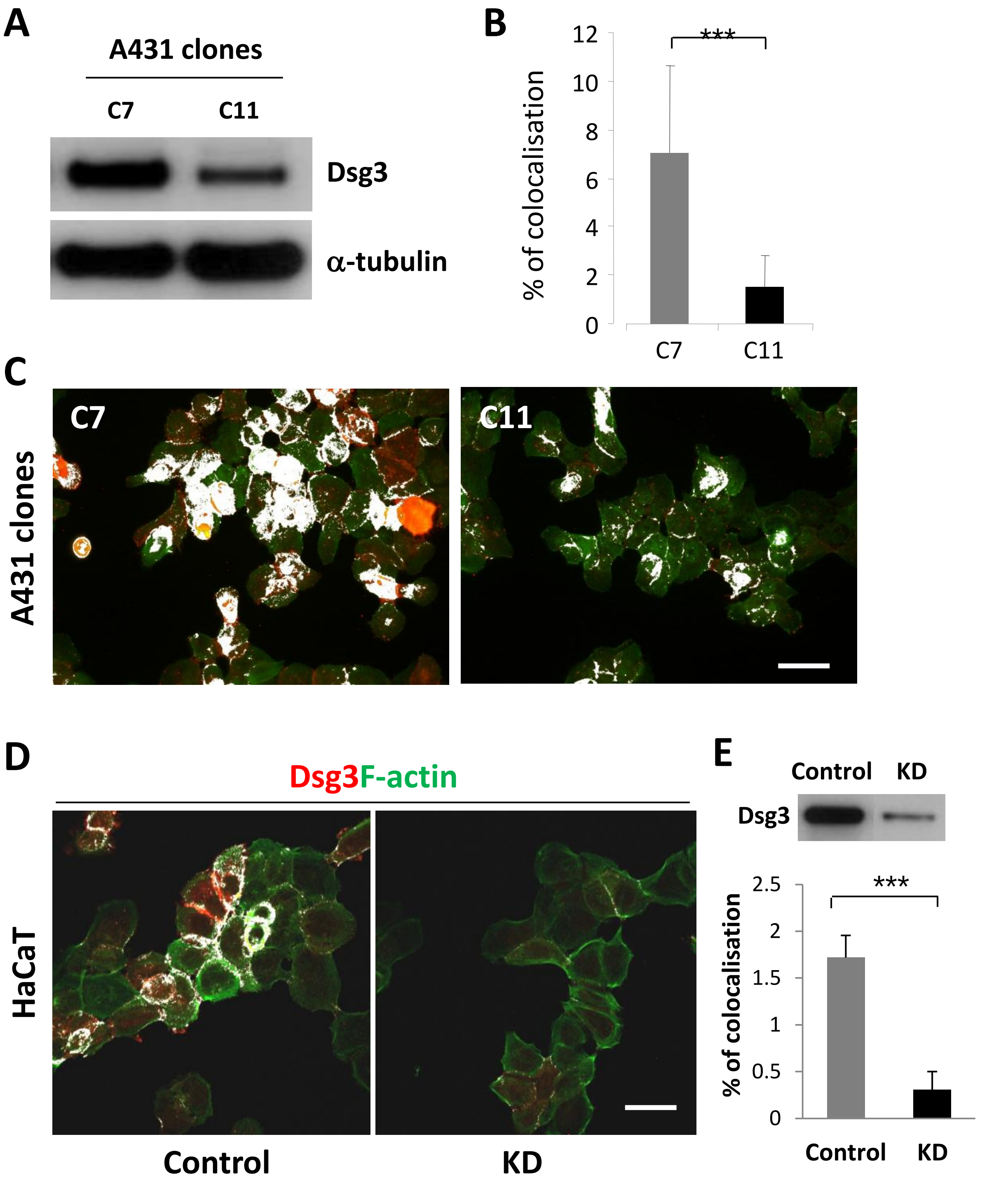

Supplementary Fig. S2 – Dose-dependent association of Dsg3 with actin in A431 cloned cells with high or low Dsg3 levels and HaCaTs with or without Dsg3 knockdown. (A) Western blotting of Dsg3 expression in C7 and C11. (B) Colocalisation analysis of Dsg3 and actin staining in C7 and C11. (C) Representative images that highlight the pixels with colocalisation of two proteins in white. (D,E) Colocalisation of Dsg3 and actin staining in HaCaTs treated with scrambled control or Dsg3 specific siRNA (KD). Significant enhanced colocalisation was seen in A431-C7 cells compared to C11 and consistently, significant reduction of the colocalisation was seen in HaCaT KD cells compared to control (mean±SEM from five arbitrary images in each group; ***$p<0.001$). Scale bars, 20µm.

Supplementary Fig. S3

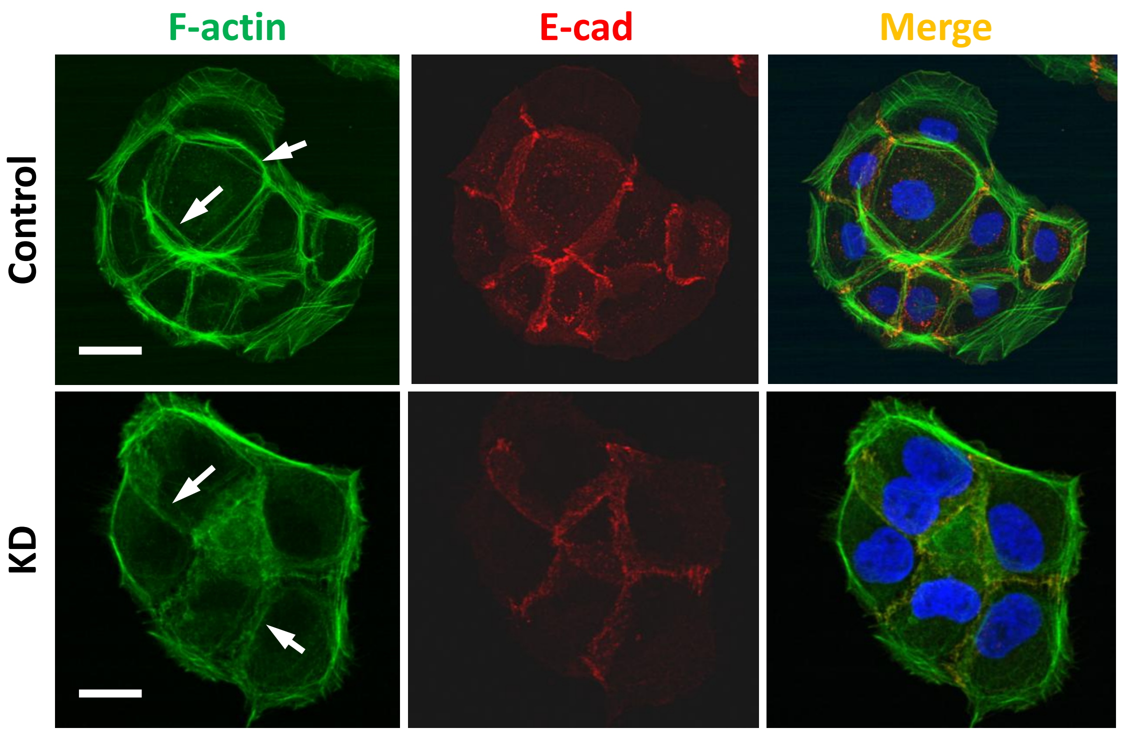

Supplementary Fig. S3 – Dsg3 is required for peripheral cortical actin assembly. Confocal images of HaCaT cells transiently transfected with scrambled control or Dsg3 specific siRNA for 48h prior to immunostaining with A488 conjugated phalloidin-actin (green) and mouse anti-E-cadherin antibody (red). Cells with Dsg3 knockdown (KD) greatly reduced both F-actin and E-cadherin staining at cell-cell junctions and caused E-cadherin distribution at the cell boarders more diffuse as compared with that in control cells. Scale bars, 10um.

Supplementary Fig. S4

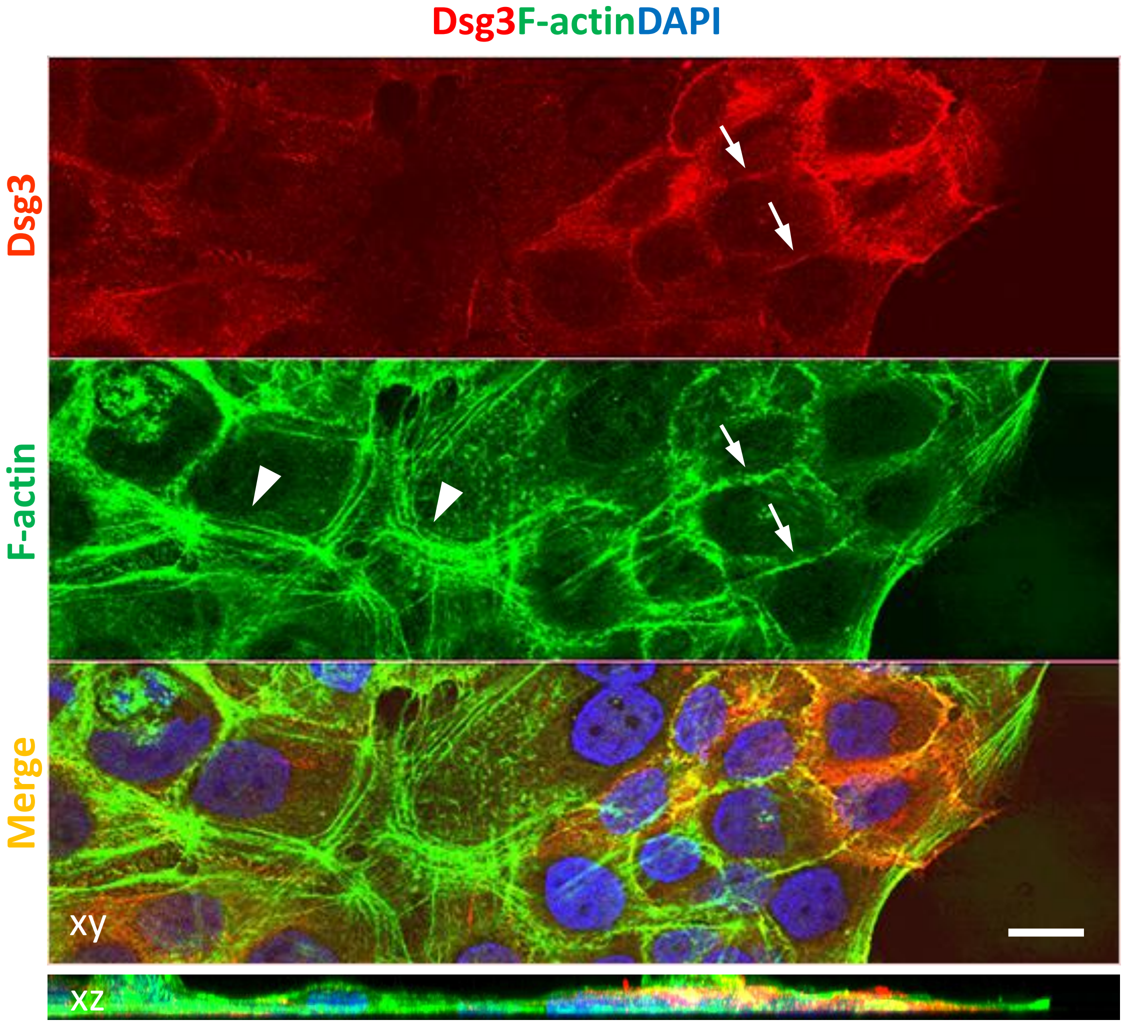

Supplementary Fig. S4 – Immunofluorescent staining of Dsg3 (red) and F-actin (green) in HaCaT cells showed that even within the same colony, cells with elevated peripheral Dsg3 expression displayed the more pronounced linear organised junctional actin (arrows) in contrast to those with low level of Dsg3 where the junctional actin bundles were depicted (arrowheads). The xz section underneath showed that cells with high levels of Dsg3 also appeared taller than those with low Dsg3 expression. Scale bars, 10um.

Supplementary Fig. S5

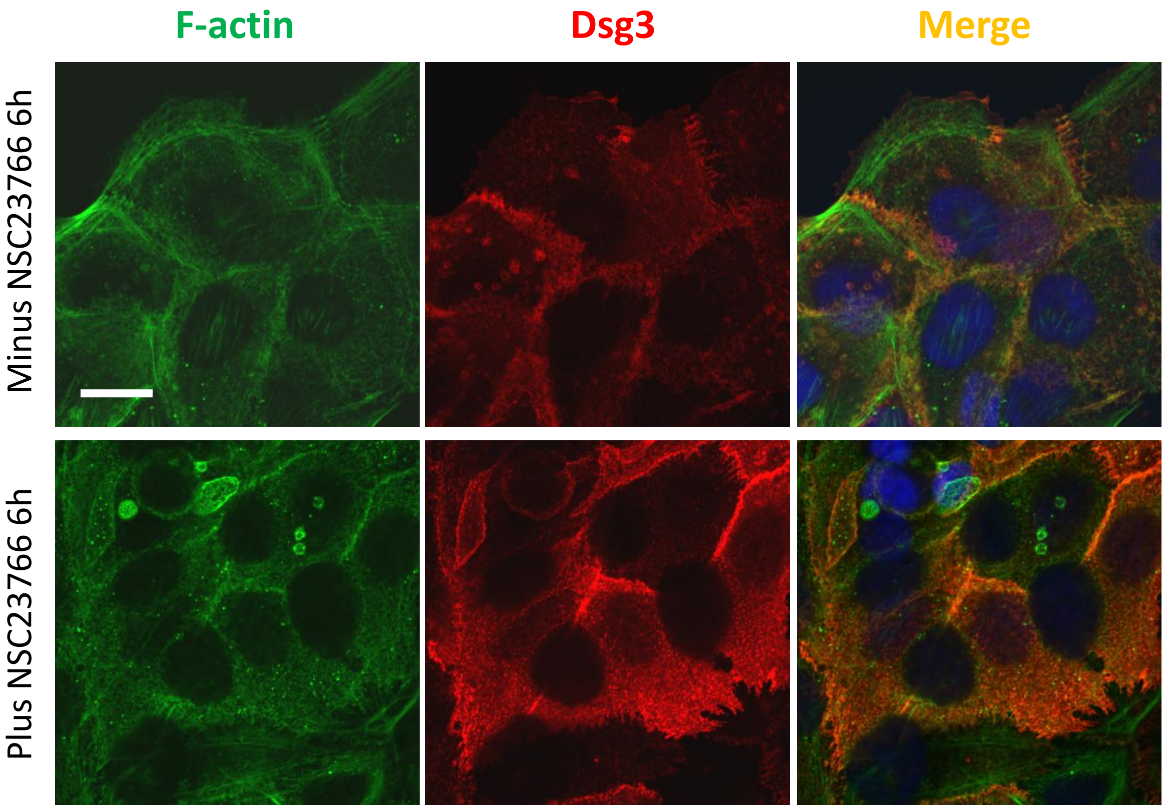

Supplementary Fig. S5 – Confocal microscopy of HaCaT cells treated in the presence and absence of the Rac inhibitor NSC23766 at a concentration of 50µM for 6 hours showed that inhibition of Rac1 caused Dsg3 immunostaining becoming more punctate at the cell boarders and also in the cytoplasm. In addition, disruption of the junctional trans-localisation and diffuse cytoplasmic immunostianing of Dsg3 were seen in the treated cells compared to control cells without drug treatment. Scale bar, 10um.
